# Conjunctival reactive cutaneous capillary endothelial proliferation induced by camrelizumab in an esophageal cancer patient: a case report and literature review

**DOI:** 10.3389/fonc.2026.1847562

**Published:** 2026-09-11

**Authors:** Yedong Wei, Ying Zan, Jing Zhang, Quan Wang, Youwei Xu, Jigang Si, Rundong Lv

**Affiliations:** 1 Department of Pharmacy, Zibo Central Hospital, Zibo, China; 2 Department of Oncology, Zibo Central Hospital, Zibo, China; 3 Department of Ophthalmology, Zibo Central Hospital, Zibo, China; 4 Department of Pharmacy, First Affiliated Hospital of Nanjing Medical University, Nanjing, China

**Keywords:** camrelizumab, case report, conjunctiva, esophageal squamous cell carcinoma, immune-related adverse event, reactive cutaneous capillary endothelial proliferation

## Abstract

**Background:**

Camrelizumab-associated reactive cutaneous capillary endothelial proliferation (RCCEP) predominantly involves cutaneous surfaces. Mucosal involvement is exceptional, with no prior reports of conjunctival lesions.

**Case presentation:**

A 64-year-old man with metastatic esophageal squamous cell carcinoma developed typical cutaneous RCCEP following initial camrelizumab exposure. Upon completion of three treatment cycles, he presented with bilateral conjunctival hyperemia, foreign body sensation, and purulent discharge. Slit-lamp examination revealed multiple vascular papules on the palpebral conjunctivae (Grade 3 RCCEP) with secondary bacterial infection. Unlike previously reported eyelid lesions requiring excision, conservative management with topical levofloxacin resulted in complete lesion resolution within four weeks without discontinuation of systemic therapy (Naranjo score: 7).

**Conclusion:**

This represents the first documented case of RCCEP affecting the conjunctival mucosa. It underscores the necessity of ophthalmologic surveillance in patients receiving camrelizumab and demonstrates that topical antimicrobial therapy constitutes an effective therapeutic strategy for complicated ocular RCCEP.

## Introduction

The awarding of the 2018 Nobel Prize in Physiology or Medicine to James P. Allison from the United States and Tasuku Honjo from Japan for their seminal contributions to tumor immunotherapy has catalyzed a global research focus on cancer immunotherapy. Significant breakthroughs have been achieved in this field, particularly with the application of immune checkpoint inhibitors (ICIs) for the treatment of various malignancies. Immune checkpoint inhibitors are primarily classified into cytotoxic T-lymphocyte antigen-4 (CTLA-4) inhibitors, programmed cell death protein-1 (PD-1) inhibitors, and inhibitors targeting its ligand, (programmed cell death protein ligand 1 (PD-L1). Camrelizumab (SHR-1210) is a PD-1 inhibitor (1) currently extensively utilized in the therapeutic management of multiple malignant tumors, including esophageal squamous cell carcinoma, Hodgkin’s lymphoma, gastric/gastroesophageal junction carcinoma, hepatocellular carcinoma, nasopharyngeal carcinoma, and non-small cell lung cancer. Camrelizumab exerts its antitumor effect by binding to PD-1 receptors expressed on T cells, thereby obstructing the interaction between PD-1 and its ligands PD-L1 or PD-L2. This blockade inhibits PD-1-mediated immunosuppressive signaling pathways (2). However, with the broadening clinical application of camrelizumab, immune-related adverse events (irAEs) associated with its administration have garnered increasing scrutiny. These adverse reactions predominantly involve organ systems such as the skin, lungs, liver, heart, and kidneys (3). Reactive cutaneous capillary endothelial proliferation (RCCEP) represents a distinctive cutaneous irAE observed following camrelizumab treatment (4). RCCEP lesions most frequently affect the head, neck, trunk, and extremities, with mucosal involvement being less common (5) and ocular region involvement considered rare. Previous literature has documented RCCEP affecting eyelid skin in a limited number of reported cases (6–9); however, conjunctival involvement remains exceptionally uncommon worldwide. Conjunctival RCCEP may precipitate complications such as hemorrhage, secondary infection, and potential visual impairment, underscoring a potential gap in clinical awareness among clinicians. This study presents a case of RCCEP with bilateral conjunctival involvement and localized infection in a patient with esophageal cancer undergoing camrelizumab therapy. A supplementary literature review and discussion are provided to offer insights for the diagnosis and clinical management of RCCEP occurring at uncommon anatomical sites.

## Case report

The patient is a 64-year-old male with a history of hypertension for over four years. His blood pressure is currently managed with oral amlodipine besylate tablets, with adequate control achieved. In July 2025, the patient developed epigastric pain without an identifiable precipitating factor, accompanied by nausea and vomiting; symptoms were alleviated postprandially. Gastroscopic examination on August 6, 2025, revealed a space-occupying lesion at the esophagogastric junction (suspected malignancy), with esophageal mucosal lesions sent for pathological evaluation. Histopathological biopsy of the esophagogastric junction on August 7, 2025, indicated poorly differentiated carcinoma, suggestive of squamous cell carcinoma. An enhanced computed tomography (CT) scan of the upper abdomen, lower abdomen, and pelvis performed on August 25, 2025, demonstrated irregular wall thickening at the lower esophageal sphincter and gastric fundus, consistent with neoplastic involvement; partial enlargement of lymph nodes in the hepatogastric space and retroperitoneum; and localized increased bone density at the sternal angle. The clinical tumor staging was cT3N2M1 (pending assessment of retroperitoneal lymph node metastasis). Given the inoperable status and absence of contraindications, antineoplastic therapy was initiated with intravenous camrelizumab (200 mg) on day 0, combined with albumin-bound paclitaxel (200 mg) on days 1 and 8, plus intravenous nedaplatin (80 mg) on days 1–2, administered in 21-day cycles. Following the first treatment cycle, the patient developed bright red punctate lesions on the trunk, head, and face, presenting as nevus-like and pearl-like macules of variable size (maximum diameter >10 mm). Some lesions on the back eroded and bled following friction but resolved spontaneously. Based on a comprehensive analysis of symptomatology, physical signs, and medical history, a diagnosis of RCCEP, Grade 2(single or multiple nodules, with the greatest nodule diameter being >10 mm, with or without rupture and bleeding), was established.

**Figure 1:**
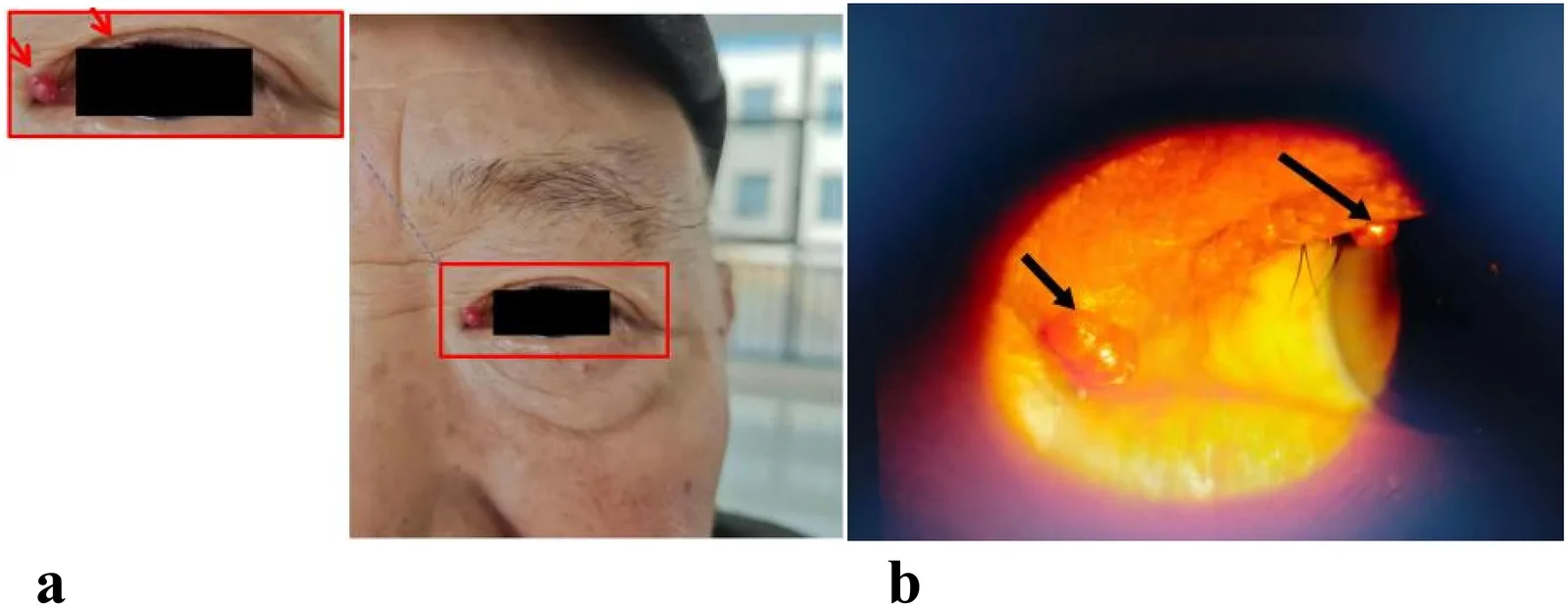
**(a)** Two red, round, elevated lesions (2×2 mm and 1×1 mm, respectively) on the left palpebral conjunctiva. **(b)** Slit-lamp photograph of the left conjunctival lesion.

**Figure 2:**
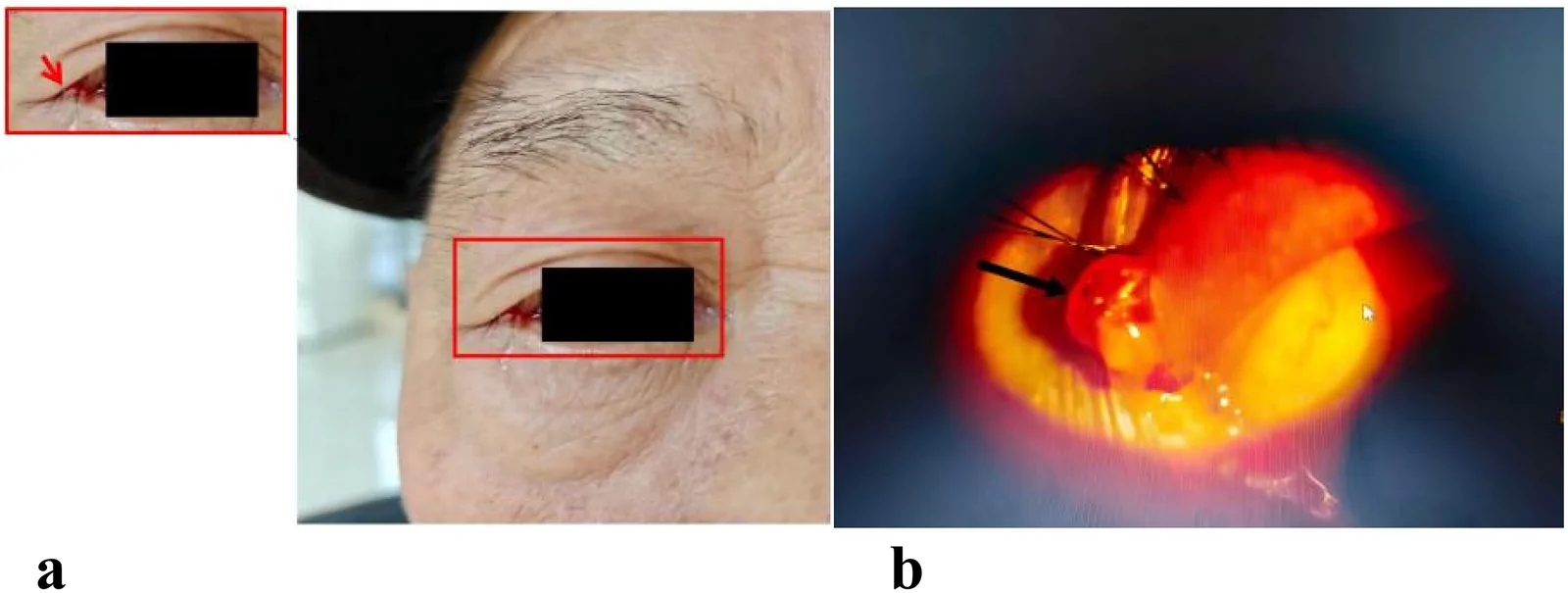
**(a)** One red, round, elevated lesions (3×3 mm) on the right palpebral conjunctiva. **(b)** Slit-lamp photograph of the right conjunctival lesion. .

**Figure 3:**
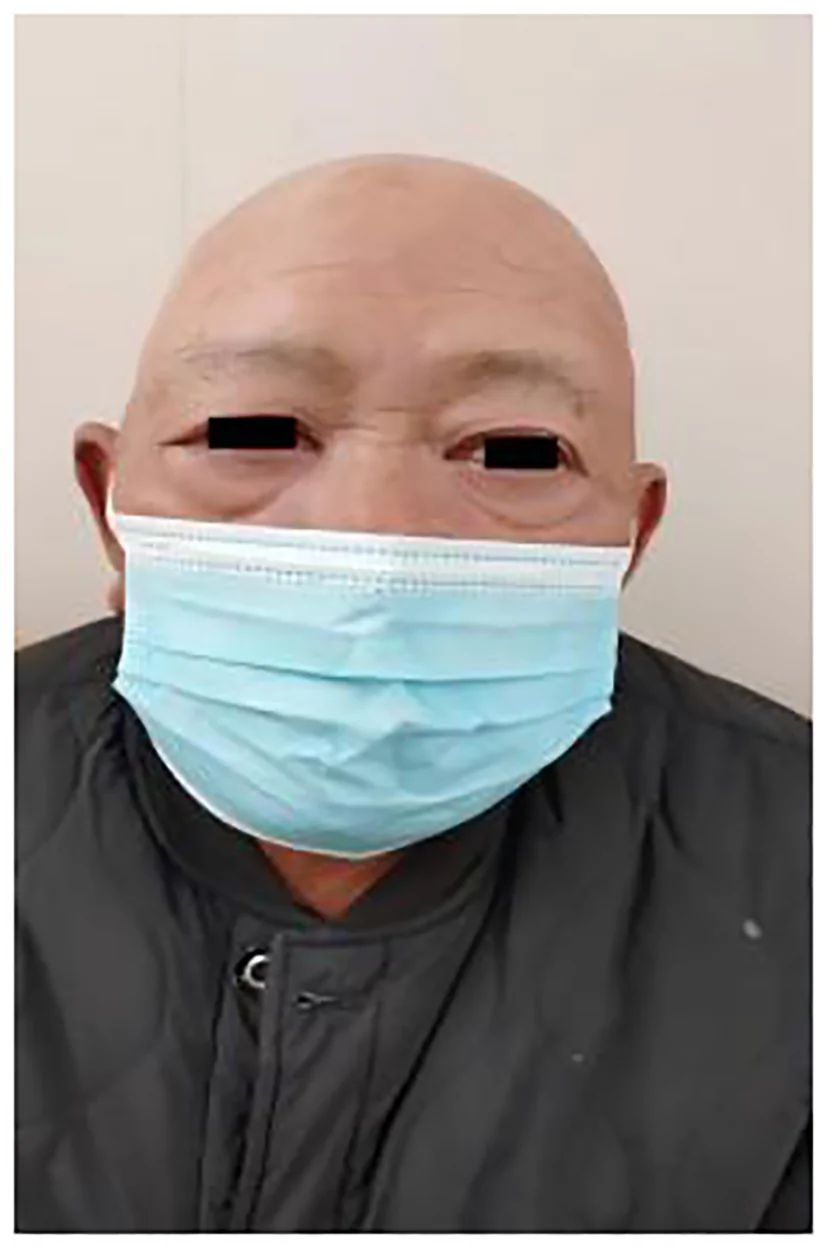
Photograph of the lesion after spontaneous detachment.

**Figure 4:**
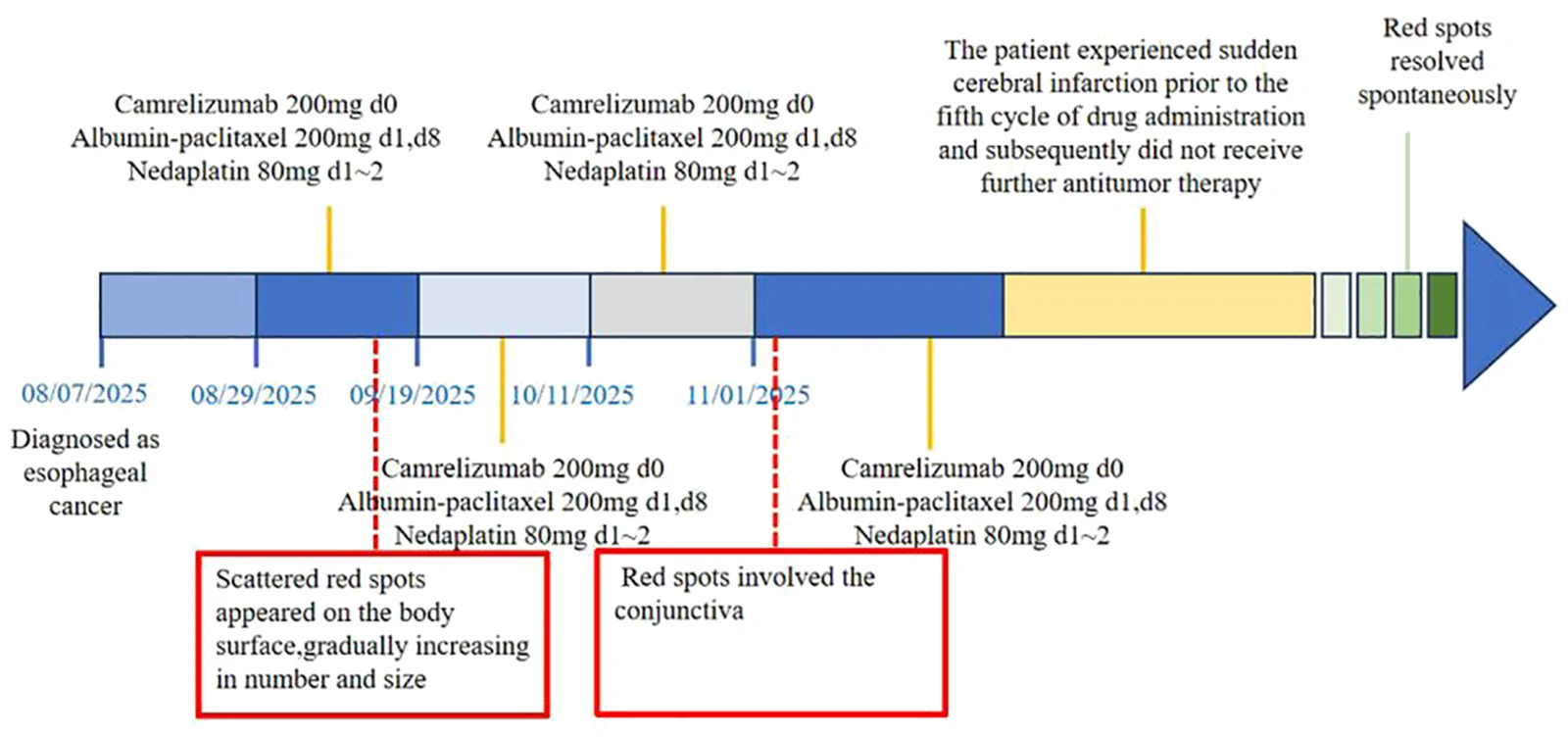
The timeline of patient progress throughout treatment.

The patient was re-admitted on November 1, 2025, for the fourth cycle of antitumor therapy. During a joint clinical round by physicians and pharmacists, the patient reported ocular foreign body sensation, pruritus, and increased secretion. Family members additionally noted ocular bleeding, suggesting probable ocular involvement of RCCEP. An ophthalmology consultation was therefore requested. Slit-lamp examination by ophthalmologists revealed bilateral palpebral conjunctival and bulbar conjunctival hyperemia, yellowish exudate in the conjunctival sac, and two red pearl-like punctate lesions (approximately 2×2 mm and 1×1 mm) on the left palpebral conjunctiva (**Figures 1a, b**), as well as a single similar lesion (approximately 3×3 mm) on the right palpebral conjunctiva (**Figures 2a, b**). The corneas were transparent with clear anterior chambers. The patient was considered to have RCCEP involving the conjunctiva, Grade 3, with subsequent ulceration, hemorrhage, and secondary local infection. Treatment was initiated with levofloxacin eye drops (one drop per eye, four times daily) bilaterally. Following appropriate administration, ocular discomfort gradually subsided and secretions decreased. The patient’s RCCEP was reduced to Grade 2 after treatment. During the fourth treatment cycle, the patient continued therapy with camrelizumab combined with albumin-bound paclitaxel and nedaplatin. No significant exacerbation of RCCEP was observed. The punctate lesion on the right palpebral conjunctiva spontaneously ruptured and bled on November 15, 2025, while the lesions on palpebral conjunctiva atrophied and sloughed off by November 29 (**Figure 3**). Punctate lesions on the head, neck, and trunk also gradually resolved. Post-treatment imaging evaluation after four cycles was planned but could not be performed because the patient developed a sudden cerebral infarction following the fourth cycle, which precluded further antitumor therapy and imaging follow-up. The patient ultimately succumbed to respiratory failure in February 2026. The timeline of the entire treatment process is illustrated in **Figure 4**.

## Discussion

### Pathogenesis of conjunctival involvement in RCCEP

The precise mechanism underlying camrelizumab-induced reactive cutaneous capillary endothelial proliferation (RCCEP) has not been fully elucidated. It has been postulated that camrelizumab, by blocking immunosuppressive pathways and reactivating immune responses, disrupts the equilibrium between pro-angiogenic and anti-angiogenic factors, ultimately leading to benign proliferation of capillary endothelial cells via pathways involving VEGF-A, FZD5, and VEGFR2 (4, 10–13). While these molecular cascades explain the general pathogenesis of RCCEP, they do not specifically account for the predilection of certain anatomical sites, such as the conjunctiva. Notably, RCCEP is observed at a substantially higher frequency with camrelizumab than with other anti-PD-1 antibodies. While the precise reasons remain unclear, several factors have been proposed, including the potential VEGFR2 agonistic activity of camrelizumab. Further studies are needed to fully elucidate the molecular basis for this distinctive adverse effect.

The conjunctiva is a translucent mucous membrane that originates at the mucocutaneous junction of the eyelid margin, covering the posterior surface of the eyelids and the anterior portion of the eyeball. It is anatomically divided into three regions: the bulbar, palpebral, and fornical conjunctiva. The palpebral conjunctiva is firmly adherent to the underlying tarsal plate, whereas the bulbar and fornical conjunctiva (except at the limbus) are loosely attached to the globe. The conjunctiva is highly vascularized and innervated, with the palpebral conjunctiva sharing its blood supply with the eyelids. Beyond its role as an ocular surface barrier, the conjunctiva contains mucosa-associated lymphoid tissue (MALT), comprising immunoglobulins, neutrophils, lymphocytes (approximately 100,000/mm²), mast cells (approximately 5,000/mm²), and plasma cells. The conjunctival stroma itself harbors antigen-presenting cells, facilitating local immune responses. Given its rich vascular network and abundant immune cell distribution, the conjunctiva represents a tissue environment particularly susceptible to immune-mediated vascular proliferative processes. This unique anatomical and immunological landscape likely explains why RCCEP can involve the palpebral conjunctiva in patients receiving camrelizumab therapy, even though the condition is primarily described as a cutaneous entity.

Additionally, it should be acknowledged that the patient was concurrently taking amlodipine for hypertension. Given its vasodilatory properties, we cannot entirely exclude the possibility that amlodipine may have contributed to the vascular proliferative process, although its specific role in RCCEP remains unclear.

### Correlation between conjunctival RCCEP and camrelizumab

Prior to camrelizumab therapy, the patient had no documented history of dermatological disorders. Before hospital admission, the patient had completed three cycles of camrelizumab in combination with chemotherapy as antitumor treatment. RCCEP emerged following the first cycle of administration. With continued treatment, RCCEP progressed to involve the conjunctiva after the third cycle. Upon cessation of camrelizumab, the patient’s RCCEP gradually resolved, demonstrating a clear temporal association between camrelizumab administration and the onset of RCCEP. The concomitant chemotherapy agents, albumin-binding paclitaxel and nedaplatin, are known to induce adverse reactions such as nausea, vomiting, bone marrow suppression, and rash; however, no cases of RCCEP have been reported in association with these drugs. Thus, the likelihood of RCCEP being induced by albumin-binding paclitaxel or nedaplatin was ruled out. Nevertheless, we cannot completely exclude the possibility that the concurrent administration of albumin-binding paclitaxel and nedaplatin might have exerted a synergistic effect on vascular endothelial proliferation or modified the phenotypic presentation of RCCEP, as chemotherapy agents are known to alter the perivascular microenvironment and may potentially lower the threshold for immune-related vascular toxicities triggered by camrelizumab. Cutaneous adverse events constitute one of the most prevalent toxicities associated with immune checkpoint inhibitors (14), while RCCEP represents the most frequently reported adverse reaction to camrelizumab (4). Based on the Naranjo’s Adverse Drug Reaction Probability Scale, the patient’s conjunctival involvement related to RCCEP scored 7, strongly indicative of camrelizumab-induced RCCEP (evaluation results are presented in **Table 1**).

**Table 1:** Naranjo’s adverse drug reaction probability scale .

Related issues	Results			Score
	Yes.	No	Not known	
1. Are there previous conclusive reports of this reaction?	+1	0	0	+1
2. Did adverse event appear after the suspected drug was given?	+2	-1	0	+2
3. Did the adverse reaction improve when the drug was discontinued or a specific antagonist was given?	+1	0	0	+1
4. Is the ADR repeated after the use of the suspected drug again?	+2	-1	0	0
5. Are there alternative causes that could have caused the reaction?	-1	+2	0	+2
6. Did the reaction reappear when a placebo was given?	-1	+1	0	0
7. Was the drug detected in any body fluid in toxic concentrations?	+1	0	0	0
8. Was the reaction more severe when the dose was increased, or less severe when the dose was decreased?	+1	0	0	+1
9. Did the patient have a similar reaction to the same or similar drugs in any previous exposure?	+1	0	0	0
10. Was the adverse event confirmed by any objective evidence?	+1	0	0	0
Total score				7

Naranjo’s score ≥ 9 points: definite, 5–8 points: probable, 1–4 points: possible, ≤ 0 points: doubtful.

### General characteristics and management of RCCEP

RCCEP primarily manifests on the cutaneous surface, with a high prevalence observed in the head and neck region, trunk, and extremities. In rare instances, it may present in areas such as the gingiva, lips, oral mucosa, ears, eyelids, breasts, colon, and nasopharynx, typically manifesting as isolated or sporadic lesions. Based on morphological features, the clinical presentations of RCCEP can be categorized into five distinct types: erythematous nevus type, pearl type, mulberry type, patchy type, and tumor-like type. Among these, the erythematous nevus type and pearl type are more frequently encountered. Histopathological examination of RCCEP nodules reveals lesions situated within the reticular dermis, characterized by proliferating capillary vessels with identifiable erythrocytes within the lumina (4). Wang et al. (4) reported that the median time to onset of camrelizumab-induced RCCEP was 4.1 weeks, with the majority of cases classified as grade 1 or 2 in severity. As the cumulative dosage increased, the nodules demonstrated a trend toward greater number and larger volume. The median disease duration was 5.6 months, with a median time to remission of 4.0 months and a median interval from the last dose to remission of 1.6 months. Most cutaneous nodules ceased growth 3–4 months following the initial dose, while some gradually regressed, desiccated, and darkened, or formed pedunculated nodules that could detach spontaneously without leaving significant scarring. RCCEP represents the most frequently reported adverse reaction associated with camrelizumab. The U.S. Food and Drug Administration (FDA) has designated RCCEP as a distinctive immune-related adverse event, with an incidence ranging from 67.0% to 79.8% when camrelizumab is administered as monotherapy (15, 16). However, phase II clinical trial data indicate that the combination of camrelizumab with apatinib substantially reduces the risk of RCCEP, with the incidence decreasing to 29.5% in patients with advanced hepatocellular carcinoma (17). Additionally, Hwang et al. (18) noted that 48.8% (40/82) of patients with advanced metastatic melanoma treated with nivolumab or pembrolizumab experienced cutaneous adverse reactions, including two cases of RCCEP (2.4%). Thus, RCCEP is not an immune-related adverse reaction exclusive to camrelizumab; other immune checkpoint inhibitors may also induce RCCEP, albeit with variations in incidence rates and clinical manifestations.

A literature review was performed using the keywords “immune checkpoint inhibitors,” “camrelizumab,” “SHR-1210,” “reactive capillary telangiectasia,” “reactive capillary hemangioma,” and “capillary telangiectasia” in the CNKI, Wanfang Medical, and PubMed databases to identify reported cases of RCCEP associated with camrelizumab from database inception through January 2026. Included cases were extracted for key clinical parameters, including patient gender, age, tumor type, treatment regimen, time to adverse reaction onset, sites of involvement, whether pathological examination of RCCEP was performed, interventions administered, and patient outcomes (**Table 2**). The inclusion criteria were as follows: (1) availability of complete case report documentation. (2) clear documentation in the literature of a causal relationship between RCCEP and camrelizumab. (3) inclusion of detailed patient demographics, medical history, and adverse event progression. Exclusion criteria comprised: (1) non-case report publications. (2) duplicate case reports. (3) incomplete medical records regarding treatment regimens or adverse reaction timelines. We compared our conjunctival case with the 44 previously reported RCCEP cases (**Table 2**). Among these, mucosal involvement was noted in 13 cases (29.5%), affecting the oral cavity (n=4), nasal cavity/nasopharynx (n=5), eyelid (n=2), gingiva (n=1), and gastrointestinal tract (n=1). Notably, conjunctival RCCEP has not been previously reported, making our case the first of its kind. In terms of severity, none of the reviewed cases reported Grade 4 or higher lesions, consistent with our patient’s Grade 3 presentation. Regarding treatment response, RCCEP at all mucosal sites—including our conjunctival case—responded favorably to either conservative management or surgical excision. This consistency suggests that conjunctival involvement, while novel, follows the generally indolent and manageable clinical trajectory characteristic of RCCEP at other anatomical sites.

**Table 2:** Summary of case reports on RCCEP induced by immune checkpoint inhibitors.

Number	Author	Publication year	Gender	Age (year)	Reason for medication	Treatment regimen	Occurrence time	Involvement sites	Histopathological examination	Intervention and outcome
1	Chen (20) et al.	2020	male	68	Polymorphic carcinoma of the lung, stage IVB	Camrelizumab 200mg, q2w	Not mentioned	Skull and trunk	no	Targeting small-molecule TKIs for VEGFR-2; RCCEP significantly reduces
2	Yu (21) et al.	2020	male	54	lung cancer	Camrelizumab 200mg, q2w	7 months after the administration of camrelizumab	gingiva	yes	Surgical excision; good healing at the surgical site
3	Long (22) et al.	2020	male	70	Squamous cell lung carcinoma	Paclitaxel + Camrelizumab 200 mg	20 days after the administration of camrelizumab	Hand	no	No specific treatment was administered; no significant changes were observed after one month.
4	Long (22) et al.	2020	female	64	Synovial sarcoma	Camrelizumab 200mg, q2w	After the first cycle of camrelizumab administration	Trunk	no	No specific treatment was administered; no significant changes were observed after one month.
5	Long (22) et al.	2020	male	54	Squamous cell lung carcinoma	Paclitaxel + Camrelizumab 200 mg	Approximately one month after the first administration of camrelizumab	Head, upper limbs, and trunk	no	No specific treatment was administered; gradual reduction was observed after one month.
6	Wei (23) et al.	2021	male	65	Suprarenal epithelioma	Camrelizumab 200 mg, q3w	After the second cycle of camrelizumab administration	Forehead, chest and back, abdomen, and limbs	no	Extend the interval between doses of camrelizumab to once every 4 weeks; gradual atrophy
7	Wei (23) et al.	2021	female	64	Adenocarcinoma of lung	Camrelizumab 200 mg, q3w	22 days after the administration of camrelizumab	Anterior neck region, head and face, thoracoabdominal region, behind the ears	no	Laser therapy; gradual reduction of skin lesions
8	Liang (24) et al.	2021	male	72	SCCL	Camrelizumab 200 mg + etoposide 160 mg/day for 3 consecutive days + cisplatin 100 mg	More than 10 days after the administration of camrelizumab	Head and face, trunk	yes	No special treatment was administered; most of the erythematous papules resolved after three months.
9	Yu (25) et al.	2021	male	55	Liver cancer	Lenvatinib + Camrelizumab 200 mg, q2w	Approximately 12 days after the first treatment with camrelizumab	Both feet and ankles, lumbar and back regions	no	No special treatment administered; gradual improvement observed
10	Zeng et al. (26)	2021	female	35	Colon cancer	Camrelizumab + Bevacizumab	15 days after treatment with camrelizumab	Skin of the chest and shoulders, mammary glands	yes	Discontinuation of medication; hemangioma shows lightening and atrophy, with bilateral breast reduction
11	Gao (27) et al.	2021	female	52	Left mandibular gingival cancer	Camrelizumab 200mg	After 2 to 3 cycles of monotherapy with camrelizumab	Abdomen (after 2 cycles) and face, head and neck, back (after 3 cycles)	no	For larger bleeding exfoliated RCCEPs, topical antibiotic application was administered; other cases did not require special treatment and were left to naturally shed.
12	Wei (28) et al.	2021	female	44	Transverse colon cancer	Camrelizumab 200 mg every 14 days + Retinoblastin 4 mg every 21 days	After 2 cycles of camrelizumab treatment	Face, neck, and chest	no	After the rupture and bleeding of the posterior neck nodule, local application of Yunnan Baiyao Capsules 0.25g bid + mupirocin ointment bid was administered, while other nodules received no special treatment; RCCEP gradually desquamated.
13	Wu (29) et al.	2022	male	57	Squamous cell carcinoma of the upper left lung lobe, stage IVB	Paclitaxel (albumin-bound) 300 mg + Camrelizumab 200 mg + Recombinant human vascular endothelin inhibitor; after one month, switch to Paclitaxel (albumin-bound) 300 mg on days 1 and 8 + Cisplatin 40 mg on days 1–3 + Camrelizumab 200 mg	More than 3 months after the first dose of camrelizumab	Skin of the face, head, neck, and chest	no	Topical application of mupirocin ointment, anlotinib 8mg qd po for 6 days; the papules became lighter in color and reduced in size.
14	Liu (30) et al.	2022	female	73	Esophageal squamous cell carcinoma	Camrelizumab 200 mg q4w + Apatinib 250 mg qd	More than 1 month after the administration of camrelizumab	Back, chest and abdomen, face, neck, ears	no	Apply Yunnan Baiyao powder topically to the chest and abdomen, and continue oral administration of apatinib 250 mg qd; after two treatment cycles, the rash subsided.
15	Wei (31) et al.	2022	male	41	Right lung adenocarcinoma, stage IVa	Ceritinib 200 mg + Pemetrexed disodium 900 mg + Carboplatin 300 mg	Day 8 after administration of camrelizumab	Left side of the waist and back	no	The treatment regimen included oral administration of loratadine tablets 10mg once daily (qd), topical application of compound lithospermum oil qd, oral administration of rhinoceros horn and rehmannia decoction tid, topical application of Kangfuxin solution qd, and oral administration of Xiaofeng powder combined with Xuanmai Ganju decoction tid. RCCEP gradually improved.
16	Zhang (32) et al.	2022	male	46	Liver cancer	Apatinib tablets 0.25g qd + Camrelizumab 200mg q21d	More than 6 months after the administration of camrelizumab	Neck, trunk	no	Oral administration of apatinib; significant regression of capillary hyperplasia
17	Zhang (33) et al.	2022	male	69	Nonsmall-cell lung cancer	Pemetrexed 0.8 g d1 + Carboplatin 400 mg d1 q3w combined with Camrelizumab 200 mg q2w	More than 3 weeks after the first administration of camrelizumab	Forehead, mandible, abdomen	yes	Surgical excision; postoperative wound healing was satisfactory
18	Zhang (33) et al.	2022	male	51	Stage IV after surgery for left lung squamous cell carcinoma	Docetaxel 100 mg d1 + cisplatin 30 mg d1–3 q2w combined with camrelizumab 200 mg q2w	Two weeks after the first administration of camrelizumab	Lower lip mucosa, chest, abdomen	yes	The lower lip mucosal mass was surgically excised; the wound healed well.
19	Hou (34) et al.	2022	male	55	Esophageal cancer	Camrelizumab 200mg	15 days after the administration of camrelizumab	Face, neck, chest and back	no	Local cryotherapy with liquid nitrogen was administered; after six months, the skin lesions showed significant reduction compared to before.
20	Wang (35) et al.	2022	male	58	Well-differentiated squamous cell carcinoma stage IV in right lung malignancy	Paclitaxel 240 mg d1 + Carboplatin 405 mg d1 q3w combined with Camrelizumab 200 mg d0	Three days after the administration of camrelizumab	Skull, face, neck, chest and abdomen	no	Oral salidomide tablets 50mg bid × 10d; the growth rate of vascular nevi was significantly slowed, with fewer new lesions appearing, and gradual spontaneous ulceration followed by crusting and desquamation.
21	Lin et al. (36)	2022	male	57	Nonkeratotic undifferentiated nasopharyngeal carcinoma, stage IVB	Camrelizumab 200 mg q3w combined with Tegafur 60 mg bid po d1~14 q3w	Camrelizumab treatment for over one month	Hands, face, and entire colon mucosa	yes	Discontinuation of camrelizumab, combined with apatinib and thalidomide therapy; improvement
22	Wang (8) et al.	2022	male	66	Suprarenal epithelioma	Sunitinib combined with camrelizumab	Approximately 2 months after the administration of camrelizumab	Head, forehead, right upper eyelid, neck, anterior chest, left palm	yes	Resection of nodules from the right upper eyelid and left palm, followed by carbon dioxide laser ablation of the remaining areas; RCCEP showed gradual improvement.
23	Wang (37) et al.	2023	female	65	Stage II serous endometrial carcinoma	Cisplatin peritoneal infusion + camrelizumab	One month after the administration of camrelizumab	Face, trunk	no	Oral thalidomide tablets 100mg once daily; complete resolution of RCCEP one month later
24	Hua (1) et al.	2023	male	59	Esophageal cancer	Reduced-dose TC regimen combined with camrelizumab 200 mg q3w	Two months after the administration of camrelizumab	Oral mucosa, head, neck, trunk, and limbs	yes	The gingival lesions and the largest facial lesion were surgically excised, with the blood supply to the pedicle of the prominent papules ligated using silk thread. All dermatological lesions resolved spontaneously and desquamated.
25	Liu (38) et al.	2023	male	67	Poorly differentiated hepatocellular carcinoma of the right hepatic lobe	Lenvatinib 12 mg qd combined with SHR-1210–200 mg q3w	Day 18 after the first injection of SHR-1210	Oral mucosa, face, trunk, and extremities	yes	Two nodules were completely resected under local anesthesia. The ruptured lesion received compression hemostasis and anti-infection treatment (fusidic acid cream applied twice daily), oral hygiene was maintained with chlorhexidine mouthwash (three times daily), and SHR-1210 was discontinued. RCCEP gradually resolved.
26	Fu (39) et al.	2023	male	52	Lung cancer	Pemetrexed + Carboplatin combined with Camrelizumab	After two treatment cycles of camrelizumab	Face, chest, and neck	no	Thalidomide 50 mg administered in the morning and 100 mg administered in the evening; gradual remission with no recurrence in RCCEP
27	Liu (5) et al.	2023	male	68	Liver cancer stage IIIb	Camrelizumab 200mg q3w	During the third dosing cycle of camrelizumab	Head, neck, trunk, and limbs	no	Oral apatinib 250 mg qd; RCCEP gradually resolved
28	Lin et al. (6)	2023	male	68	Nasopharyngeal carcinoma T3N2M1	Capecitabine 625 mg/m² bid + Camrelizumab 200 mg q21d	After the seventh cycle of treatment with camrelizumab	Head, face, trunk, eyelids	yes	The right lower eyelid nodule was surgically excised; postoperative eyelid recovery was satisfactory, and other RCCEPs resolved spontaneously.
29	Yao (40) et al.	2023	male	54	Duodenal papillary carcinoma	Camrelizumab 200mg q3w	Day 17 of first administration of camrelizumab	Face, neck, trunk, and limbs	yes	For larger masses with partial infiltration, CO2 laser therapy was administered after local anesthesia, followed by topical application of Yunnan Baiyao and compression hemostasis at the ulcerated bleeding site. Most nodules exhibited atrophy and regression.
30	Xu et al. (41)	2023	female	64	Esophageal squamous cell carcinoma	Capecitabine orally 1g bid d1–14 q3w + Camrelizumab 200mg q3w	After the fourth cycle of camrelizumab treatment	Neck, face, lips, nail grooves of fingers	no	Sclerotherapy of the hemorrhagic tumor mass using polidocanol, surgical excision of the nail fold nodule; progressive resolution of RCCEP
31	Huang (42) et al.	2023	male	69	Carcinoma of sigmoid	Camrelizumab 200mg	During the third cycle of camrelizumab administration	Head, face, neck, shoulders, back, right zygomatic region	no	A large mass in the zygomatic region was surgically excised, with no further special treatment applied to the remaining area; RCCEP showed gradual improvement.
32	Gu (43) et al.	2023	female	42	Adenocarcinoma of the right half of the colon, stage III	After 17 months of treatment with FOLFIRI combined with camrelizumab 200 mg, the regimen was switched to camrelizumab monotherapy 200 mg every 21 days.	First application of camrelizumab over six months ago	Face, neck, back, and trunk	no	Ceritinib was suspended, and empirical anti-infective therapy with cefazolin was administered. Topical mupirocin was used for anti-infective treatment. The larger vascular nevus underwent surgical excision, followed by topical application of Yunnan Baiyao for hemostasis postoperatively. The patient’s infection indicators gradually declined, the vascular nevus progressively darkened and reduced in size, and symptoms showed gradual improvement.
33	Yu (9) et al.	2023	male	67	Right lung adenocarcinoma, stage IV	Pemetrexed + Carboplatin combined with Camrelizumab 200 mg	One cycle of camrelizumab administration	Nose tip, right upper eyelid, scalp area	no	The vascular nevus gradually darkens in color.
34	Yu (9) et al.	2023	male	75	Left lung squamous cell carcinoma, stage IIB	Paclitaxel (albumin-bound) + Carboplatin combined with camrelizumab 200 mg	Camrelizumab administration for over one month	Face and trunk	no	No specific treatment was administered; new vascular nevus was observed during follow-up two months later.
35	Jie (44) et al.	2023	female	50	Left lung adenocarcinoma, stage IVB	Pemetrexed 786 mg d1 combined with camrelizumab 200 mg d1	After the second cycle of camrelizumab treatment	Left breast	yes	No specific treatment was administered; RCCEP resolved spontaneously.
36	Hui (45) et al.	2024	female	49	Acute myeloid leukemia type M2	Camrelizumab 200mg	13 days after administration of camrelizumab	Neck, chest, and back	no	No specific treatment was administered; RCCEP resolved spontaneously.
37	Zhou (7) et al.	2024	female	44	Cervical carcinoma	SHR-1210 200mg q3w	One week after initial administration of SHR-1210 treatment	Eyelid margin, forehead, and scalp	yes	No specific treatment was administered; RCCEP resolved spontaneously.
38	Zheng (46) et al.	2024	female	58	Cervical squamous cell carcinoma stage III	Camrelizumab 200mg	Approximately 3 weeks after the first injection of camrelizumab	Head and face, lower lip, trunk, and limbs	yes	Camrelizumab was discontinued, and the lip nodules were surgically excised with observation of remaining skin lesions; two months after discontinuation, the lesions began to regress spontaneously.
39	Tan (47) et al.	2025	male	50	Stage IV gastric cancer	Camrelizumab 200 mg d1 + albumin-bound paclitaxel 200 mg d1, d8, q21d, chemotherapy combined with immunotherapy for 7 cycles; subsequently switched to furquintinib 3 mg qd + camrelizumab 200 mg d1 for combined therapy for 9 cycles.	After 18 cycles of camrelizumab administration	Ascending colon, transverse colon, descending colon	no	Ceritinib was suspended, and methylprednisolone was administered for anti-inflammatory treatment, along with compound glutamine to repair intestinal mucosa and paracetamol-dihydrocodeine tablets for analgesia. The patient’s abdominal pain alleviated without recurrence.
40	Huang (48) et al.	2025	male	61	Primary liver cancer	Apatinib combined with camrelizumab	After 9 cycles of camrelizumab administration	Concha nasalis inferior	yes	Local anesthesia was used for surgical resection; follow-up for 12 months showed no recurrence.
41	Huang (48) et al.	2025	male	31	Nasopharyngeal darcinoma	Albumin-bound paclitaxel combined with camrelizumab	After 9 cycles of camrelizumab administration	Pars nasalis pharyngis	yes	General anesthesia was used for surgical resection; follow-up for 12 months showed no recurrence.
42	Huang (48) et al.	2025	female	28	Nasopharyngeal darcinoma	Nintopuzumab + albumin-bound paclitaxel + cisplatin combined with camrelizumab	After 15 cycles of camrelizumab administration	Pars nasalis pharyngis	yes	General anesthesia was used for surgical resection; follow-up for 12 months showed no recurrence.
43	Huang (48) et al.	2025	female	38	Nasopharyngeal darcinoma	Camrelizumab, combination therapy with unknown drugs	After two cycles of camrelizumab administration	Nasal septum	yes	General anesthesia was used for surgical resection; follow-up for 6 months showed no recurrence.
44	Huang (48) et al.	2025	male	21	Nasopharyngeal darcinoma	Docetaxel + cisplatin combined with camrelizumab	After 14 cycles of camrelizumab administration	Nasal septum, nasal floor	yes	General anesthesia was used for surgical resection; follow-up for 8 months showed no recurrence.

Based on clinical research and observational evidence, RCCEP can be categorized into five distinct grades (19), with the corresponding clinical manifestations and recommended management strategies for each grade outlined in **Table 3**. The grading criteria presented in **Table 3** are adapted from the expert consensus on camrelizumab-induced RCCEP. Although this consensus was primarily developed for cutaneous RCCEP, it explicitly acknowledges that RCCEP may occasionally involve mucosal sites, including the conjunctiva. Given the absence of a site-specific guideline for conjunctival involvement, and considering the shared vascular and immunological features between the conjunctiva and skin, we considered this consensus to be the most appropriate reference for grading and managing our patient’s conjunctival lesion. The grading criteria for RCCEP are as follows: Grade 1: Single or multiple nodules, with the largest diameter ≤ 10 mm, with or without ulceration/bleeding; Grade 2: Single or multiple nodules, with the largest diameter > 10 mm, with or without ulceration/bleeding; Grade 3: Generalized skin nodules involving the entire body, complicated by skin infection; Grade 4: Multiple or generalized lesions, life-threatening; Grade 5: Death. According to the RCCEP grading criteria, drug discontinuation are generally recommended for Grade 3 or higher lesions with evidence of generalized infection or life-threatening complications. In our case, given the patient’s explicit refusal of surgical excision and the favorable antitumor response to camrelizumab, we opted for a conservative approach with topical antibiotic therapy to manage secondary superficial infection. This decision successfully reduced the lesion from Grade 3 to Grade 2, enabling continued immunotherapy without compromising treatment efficacy.

**Table 3:** RCCEP Grading Criteria and Treatment Recommendations.

Classify	Clinical manifestation	Treatment recommendation
Level 1	Single or multiple nodules with a maximum diameter ≤10 mm, with or without ulceration and bleeding	Continue medication administration. Apply gauze protection to areas prone to friction to prevent bleeding. For cases with ulceration and bleeding, local compression therapy may be employed for hemostasis.
Level 2	Single or multiple nodules with a maximum diameter>10 mm, with or without ulceration and bleeding	Continue medication administration. Apply gauze protection to areas prone to friction to prevent bleeding. For ulcerated bleeding, local compression hemostasis may be employed, or local therapeutic interventions such as laser therapy or surgical excision may be considered. Prevent infection at the ulcerated site.
Level 3	The condition is generalized and may be complicated by infection, potentially requiring hospitalization.	Discontinue medication until the condition is restored to grade ≤1 before resuming administration. Apply gauze protection to areas prone to friction to prevent bleeding. For cases with ulceration and bleeding, local compression may be employed for hemostasis, or local therapeutic interventions such as laser therapy or surgical excision may be considered. Concurrent infections require antimicrobial therapy.
Level 4	Multiple and generalized manifestations, life-threatening	Discontinue medication permanently and seek immediate medical attention
Level 5	die	

### Regarding the patient’s cause of death

The patient ultimately died of sudden cerebral infarction complicated by respiratory failure. It is important to acknowledge that immune checkpoint inhibitors (ICIs), including camrelizumab, have been increasingly associated with a spectrum of immune-related adverse events (irAEs), among which cerebrovascular events such as ischemic stroke have been reported in a growing number of cases. In our patient, the presence of pre-existing cerebrovascular risk factors—namely advanced age, a history of prior cerebral infarction prior to immunotherapy, and an active malignancy—makes causal attribution particularly challenging. The neurology consultation and cranial MRI findings confirmed acute-to-subacute infarction in the left cerebral hemisphere with multiple ischemic foci; however, the enhancing lesions in the left frontal lobe could not definitively exclude the possibility of brain metastases. While the treating team considered the cerebral infarction to be most likely multifactorial in origin, we cannot entirely exclude the possibility that camrelizumab therapy may have contributed to the cerebrovascular event, either through direct immune-mediated vascular injury or via an indirect pro-inflammatory effect. Unfortunately, additional workup (e.g., vascular imaging, autoimmune serologies, or lymphocyte subset analysis) could not be performed due to the patient’s rapid clinical deterioration. This case highlights the need for heightened clinical vigilance for cerebrovascular complications in patients receiving ICI therapy, particularly those with pre-existing vascular risk factors.

## Limitations

It is important to acknowledge that the literature summary presented in **Table 2** was conducted as a non-systematic narrative literature review, rather than a formal systematic review or meta-analysis. Therefore, this summary does not strictly adhere to the PRISMA guidelines for systematic reviews. Specifically, we did not employ a structured, repeatable search strategy with a detailed combination of Boolean operators across multiple databases, nor did we perform formal risk-of-bias assessments or quality scoring for the included case reports. Consequently, the data extracted from these reports are preliminary and may be subject to selection bias or incomplete retrieval of relevant cases. Our literature search covered publications up to January 2026, and we have stated this cut-off date clearly in this section. As this was not a PRISMA-compliant systematic review, we did not perform a formal study selection flow diagram or quality assessment scoring. Nevertheless, the primary goal of this narrative summary was to provide a snapshot of the currently documented clinical characteristics of RCCEP to contextualize our index case, rather than to provide a pooled quantitative estimate of incidence or treatment outcomes.

The absence of post-treatment imaging evaluation represents a limitation of this case; however, it also reflects the clinical reality that severe immune-related adverse events may interrupt otherwise effective treatment regimens.

## Conclusion

In summary, immune checkpoint inhibitors have achieved remarkable progress in the treatment of malignant tumors, heralding a new era in cancer immunotherapy; however, they have also been associated with numerous immune-related adverse events. RCCEP constitutes the most prevalent cutaneous immune-related adverse reaction induced by camrelizumab, predominantly manifesting in the head and neck region, trunk, and extremities. It is characterized by distinctive morphological features that demonstrate dynamic evolution and self-limiting properties. This report presents a case of RCCEP involving the bilateral eyelid conjunctiva in a patient with esophageal squamous cell carcinoma following camrelizumab administration. Ocular involvement of RCCEP, particularly in the eyelid conjunctival region, is exceedingly rare and necessitates a comprehensive diagnostic and differential diagnosis based on patient history. This case underscores the critical importance of recognizing the clinical manifestations of camrelizumab-induced RCCEP during treatment, conducting routine ocular examinations before and after drug administration, enabling early detection of ocular abnormalities, and providing appropriate symptomatic management to avert missed or misdiagnosed cases.

## Data Availability

The datasets presented in this study can be found in online repositories. The names of the repository/repositories and accession number(s) can be found in the article/**Supplementary Material**.
