# Supplementary material for: Conjunctival reactive cutaneous capillary endothelial proliferation induced by camrelizumab in an esophageal cancer patient: a case report and literature review
Source: Front Oncol. 2026 Sep 11;16:1847562. doi: 10.3389/fonc.2026.1847562 (PMC13612280; doi:10.3389/fonc.2026.1847562)
Supplement: Supplementary file 1 [file SupplementaryFile1.docx]

Supplementary Material

# 1 Supplementary Figures

Figure1a: Two red, round, elevated lesions (2×2 mm and 1×1 mm, respectively) on the left palpebral conjunctiva.

Figure1b: Slit-lamp photograph of the left conjunctival lesion.

Figure2a: One red, round, elevated lesions (3×3 mm ) on the right palpebral conjunctiva.

Figure2b: Slit-lamp photograph of the right conjunctival lesion.

Figure3: Photograph of the lesion after spontaneous detachment.

Figure4: The timeline of patient progress throughout treatment.

# 2 Supplementary Tables

**Table 1: Naranjo’s adverse drug reaction probability scale**

| Related issues | Results | | | Score |
| --- | --- | --- | --- | --- |
|  | Yes. | No | Not known |  |
| 1. Are there previous conclusive reports of this reaction? | +1 | 0 | 0 | +1 |
| 2. Did adverse event appear after the suspected drug was given? | +2 | -1 | 0 | +2 |
| 3. Did the adverse reaction improve when the drug was discontinued or a specific antagonist was given? | +1 | 0 | 0 | +1 |
| 4. Is the ADR repeated after the use of the suspected drug again? | +2 | -1 | 0 | 0 |
| 5. Are there alternative causes that could have caused the reaction? | -1 | +2 | 0 | +2 |
| 6. Did the reaction reappear when a placebo was given? | -1 | +1 | 0 | 0 |
| 7. Was the drug detected in any body fluid in toxic concentrations? | +1 | 0 | 0 | 0 |
| 8. Was the reaction more severe when the dose was increased, or less severe when the dose was decreased? | +1 | 0 | 0 | +1 |
| 9. Did the patient have a similar reaction to the same or similar drugs in any previous exposure? | +1 | 0 | 0 | 0 |
| 10. Was the adverse event confirmed by any objective evidence? | +1 | 0 | 0 | 0 |
| Total score |  |  |  | 7 |

Naranjo’s score ≥ 9 points: definite, 5-8 points: probable, 1-4 points: possible, ≤ 0 points: doubtful.

**Table 2 Summary of case reports on RCCEP induced by immune checkpoint inhibitors**

| Number | Author | Publication year | Gender | Age  （ year ） | Reason for medication | Treatment regimen | Occurrence time | Involvement sites | Histopathological examination | Intervention and outcome |
| --- | --- | --- | --- | --- | --- | --- | --- | --- | --- | --- |
| 1 | Chen[20] et al. | 2020 | male | 68 | Polymorphic carcinoma of the lung, stage IVB | Camrelizumab 200mg, q2w | Not mentioned | Skull and trunk | no | Targeting small-molecule TKIs for VEGFR-2; RCCEP significantly reduces |
| 2 | Yu [21] et al. | 2020 | male | 54 | lung cancer | Camrelizumab 200mg, q2w | 7 months after the administration of camrelizumab | gingiva | yes | Surgical excision; good healing at the surgical site |
| 3 | Long[22] et al. | 2020 | male | 70 | Squamous cell lung carcinoma | Paclitaxel + Camrelizumab 200 mg | 20 days after the administration of camrelizumab | Hand | no | No specific treatment was administered; no significant changes were observed after one month. |
| 4 | Long[22] et al. | 2020 | female | 64 | Synovial sarcoma | Camrelizumab 200mg, q2w | After the first cycle of camrelizumab administration | Trunk | no | No specific treatment was administered; no significant changes were observed after one month. |
| 5 | Long[22] et al. | 2020 | male | 54 | Squamous cell lung carcinoma | Paclitaxel + Camrelizumab 200 mg | Approximately one month after the first administration of camrelizumab | Head, upper limbs, and trunk | no | No specific treatment was administered; gradual reduction was observed after one month. |
| 6 | Wei [23] et al. | 2021 | male | 65 | Suprarenal epithelioma | Camrelizumab 200 mg, q3w | After the second cycle of camrelizumab administration | Forehead, chest and back, abdomen, and limbs | no | Extend the interval between doses of camrelizumab to once every 4 weeks; gradual atrophy |
| 7 | Wei [23] et al. | 2021 | female | 64 | Adenocarcinoma of lung | Camrelizumab 200 mg, q3w | 22 days after the administration of camrelizumab | Anterior neck region, head and face, thoracoabdominal region, behind the ears | no | Laser therapy; gradual reduction of skin lesions |
| 8 | Liang[24] et al. | 2021 | male | 72 | SCCL | Camrelizumab 200 mg + etoposide 160 mg/day for 3 consecutive days + cisplatin 100 mg | More than 10 days after the administration of camrelizumab | Head and face, trunk | yes | No special treatment was administered; most of the erythematous papules resolved after three months. |
| 9 | Yu [25] et al. | 2021 | male | 55 | Liver cancer | Lenvatinib + Camrelizumab 200 mg, q2w | Approximately 12 days after the first treatment with camrelizumab | Both feet and ankles, lumbar and back regions | no | No special treatment administered; gradual improvement observed |
| 10 | Zeng et al. [26] | 2021 | female | 35 | Colon cancer | Camrelizumab + Bevacizumab | 15 days after treatment with camrelizumab | Skin of the chest and shoulders, mammary glands | yes | Discontinuation of medication; hemangioma shows lightening and atrophy, with bilateral breast reduction |
| 11 | Gao[27] et al. | 2021 | female | 52 | Left mandibular gingival cancer | Camrelizumab 200mg | After 2 to 3 cycles of monotherapy with camrelizumab | Abdomen (after 2 cycles) and face, head and neck, back (after 3 cycles) | no | For larger bleeding exfoliated RCCEPs, topical antibiotic application was administered; other cases did not require special treatment and were left to naturally shed. |
| 12 | Wei [28] et al. | 2021 | female | 44 | Transverse colon cancer | Camrelizumab 200 mg every 14 days + Retinoblastin 4 mg every 21 days | After 2 cycles of camrelizumab treatment | Face, neck, and chest | no | After the rupture and bleeding of the posterior neck nodule, local application of Yunnan Baiyao Capsules 0.25g bid + mupirocin ointment bid was administered, while other nodules received no special treatment; RCCEP gradually desquamated. |
| 13 | Wu [29] et al. | 2022 | male | 57 | Squamous cell carcinoma of the upper left lung lobe, stage IVB | Paclitaxel (albumin-bound) 300 mg + Camrelizumab 200 mg + Recombinant human vascular endothelin inhibitor; after one month, switch to Paclitaxel (albumin-bound) 300 mg on days 1 and 8 + Cisplatin 40 mg on days 1–3 + Camrelizumab 200 mg | More than 3 months after the first dose of camrelizumab | Skin of the face, head, neck, and chest | no | Topical application of mupirocin ointment, anlotinib 8mg qd po for 6 days; the papules became lighter in color and reduced in size. |
| 14 | Liu[30] et al. | 2022 | female | 73 | Esophageal squamous cell carcinoma | Camrelizumab 200 mg q4w + Apatinib 250 mg qd | More than 1 month after the administration of camrelizumab | Back, chest and abdomen, face, neck, ears | no | Apply Yunnan Baiyao powder topically to the chest and abdomen, and continue oral administration of apatinib 250 mg qd; after two treatment cycles, the rash subsided. |
| 15 | Wei [31] et al. | 2022 | male | 41 | Right lung adenocarcinoma, stage IVa | Ceritinib 200 mg + Pemetrexed disodium 900 mg + Carboplatin 300 mg | Day 8 after administration of camrelizumab | Left side of the waist and back | no | The treatment regimen included oral administration of loratadine tablets 10mg once daily (qd), topical application of compound lithospermum oil qd, oral administration of rhinoceros horn and rehmannia decoction tid, topical application of Kangfuxin solution qd, and oral administration of Xiaofeng powder combined with Xuanmai Ganju decoction tid. RCCEP gradually improved. |
| 16 | Zhang[32] et al. | 2022 | male | 46 | Liver cancer | Apatinib tablets 0.25g qd + Camrelizumab 200mg q21d | More than 6 months after the administration of camrelizumab | Neck, trunk | no | Oral administration of apatinib; significant regression of capillary hyperplasia |
| 17 | Zhang[33] et al. | 2022 | male | 69 | Nonsmall-cell lung cancer | Pemetrexed 0.8 g d1 + Carboplatin 400 mg d1 q3w combined with Camrelizumab 200 mg q2w | More than 3 weeks after the first administration of camrelizumab | Forehead, mandible, abdomen | yes | Surgical excision; postoperative wound healing was satisfactory |
| 18 | Zhang[33] et al. | 2022 | male | 51 | Stage IV after surgery for left lung squamous cell carcinoma | Docetaxel 100 mg d1 + cisplatin 30 mg d1–3 q2w combined with camrelizumab 200 mg q2w | Two weeks after the first administration of camrelizumab | Lower lip mucosa, chest, abdomen | yes | The lower lip mucosal mass was surgically excised; the wound healed well. |
| 19 | Hou[34] et al. | 2022 | male | 55 | Esophageal cancer | Camrelizumab 200mg | 15 days after the administration of camrelizumab | Face, neck, chest and back | no | Local cryotherapy with liquid nitrogen was administered; after six months, the skin lesions showed significant reduction compared to before. |
| 20 | Wang [35] et al. | 2022 | male | 58 | Well-differentiated squamous cell carcinoma stage IV in right lung malignancy | Paclitaxel 240 mg d1 + Carboplatin 405 mg d1 q3w combined with Camrelizumab 200 mg d0 | Three days after the administration of camrelizumab | Skull, face, neck, chest and abdomen | no | Oral salidomide tablets 50mg bid × 10d; the growth rate of vascular nevi was significantly slowed, with fewer new lesions appearing, and gradual spontaneous ulceration followed by crusting and desquamation. |
| 21 | Lin et al. [36] | 2022 | male | 57 | Nonkeratotic undifferentiated nasopharyngeal carcinoma, stage IVB | Camrelizumab 200 mg q3w combined with Tegafur 60 mg bid po d1~14 q3w | Camrelizumab treatment for over one month | Hands, face, and entire colon mucosa | yes | Discontinuation of camrelizumab, combined with apatinib and thalidomide therapy; improvement |
| 22 | Wang [8] et al. | 2022 | male | 66 | Suprarenal epithelioma | Sunitinib combined with camrelizumab | Approximately 2 months after the administration of camrelizumab | Head, forehead, right upper eyelid, neck, anterior chest, left palm | yes | Resection of nodules from the right upper eyelid and left palm, followed by carbon dioxide laser ablation of the remaining areas; RCCEP showed gradual improvement. |
| 23 | Wang [37] et al. | 2023 | female | 65 | Stage II serous endometrial carcinoma | Cisplatin peritoneal infusion + camrelizumab | One month after the administration of camrelizumab | Face, trunk | no | Oral thalidomide tablets 100mg once daily; complete resolution of RCCEP one month later |
| 24 | Hua[1] et al. | 2023 | male | 59 | Esophageal cancer | Reduced-dose TC regimen combined with camrelizumab 200 mg q3w | Two months after the administration of camrelizumab | Oral mucosa, head, neck, trunk, and limbs | yes | The gingival lesions and the largest facial lesion were surgically excised, with the blood supply to the pedicle of the prominent papules ligated using silk thread. All dermatological lesions resolved spontaneously and desquamated. |
| 25 | Liu[38] et al. | 2023 | male | 67 | Poorly differentiated hepatocellular carcinoma of the right hepatic lobe | Lenvatinib 12 mg qd combined with SHR-1210 200 mg q3w | Day 18 after the first injection of SHR-1210 | Oral mucosa, face, trunk, and extremities | yes | Two nodules were completely resected under local anesthesia. The ruptured lesion received compression hemostasis and anti-infection treatment (fusidic acid cream applied twice daily), oral hygiene was maintained with chlorhexidine mouthwash (three times daily), and SHR-1210 was discontinued. RCCEP gradually resolved. |
| 26 | Fu[39] et al. | 2023 | male | 52 | Lung cancer | Pemetrexed + Carboplatin combined with Camrelizumab | After two treatment cycles of camrelizumab | Face, chest, and neck | no | Thalidomide 50 mg administered in the morning and 100 mg administered in the evening; gradual remission with no recurrence in RCCEP |
| 27 | Liu[5] et al. | 2023 | male | 68 | Liver cancer stage IIIb | Camrelizumab 200mg q3w | During the third dosing cycle of camrelizumab | Head, neck, trunk, and limbs | no | Oral apatinib 250 mg qd; RCCEP gradually resolved |
| 28 | Lin et al. [6] | 2023 | male | 68 | Nasopharyngeal carcinoma T3N2M1 | Capecitabine 625 mg/m² bid + Camrelizumab 200 mg q21d | After the seventh cycle of treatment with camrelizumab | Head, face, trunk, eyelids | yes | The right lower eyelid nodule was surgically excised; postoperative eyelid recovery was satisfactory, and other RCCEPs resolved spontaneously. |
| 29 | Yao[40] et al. | 2023 | male | 54 | Duodenal papillary carcinoma | Camrelizumab 200mg q3w | Day 17 of first administration of camrelizumab | Face, neck, trunk, and limbs | yes | For larger masses with partial infiltration, CO2 laser therapy was administered after local anesthesia, followed by topical application of Yunnan Baiyao and compression hemostasis at the ulcerated bleeding site. Most nodules exhibited atrophy and regression. |
| 30 | Xu et al. [41] | 2023 | female | 64 | Esophageal squamous cell carcinoma | Capecitabine orally 1g bid d1-14 q3w + Camrelizumab 200mg q3w | After the fourth cycle of camrelizumab treatment | Neck, face, lips, nail grooves of fingers | no | Sclerotherapy of the hemorrhagic tumor mass using polidocanol, surgical excision of the nail fold nodule; progressive resolution of RCCEP |
| 31 | Huang[42] et al. | 2023 | male | 69 | Carcinoma of sigmoid | Camrelizumab 200mg | During the third cycle of camrelizumab administration | Head, face, neck, shoulders, back, right zygomatic region | no | A large mass in the zygomatic region was surgically excised, with no further special treatment applied to the remaining area; RCCEP showed gradual improvement. |
| 32 | Gu[43] et al. | 2023 | female | 42 | Adenocarcinoma of the right half of the colon, stage III | After 17 months of treatment with FOLFIRI combined with camrelizumab 200 mg, the regimen was switched to camrelizumab monotherapy 200 mg every 21 days. | First application of camrelizumab over six months ago | Face, neck, back, and trunk | no | Ceritinib was suspended, and empirical anti-infective therapy with cefazolin was administered. Topical mupirocin was used for anti-infective treatment. The larger vascular nevus underwent surgical excision, followed by topical application of Yunnan Baiyao for hemostasis postoperatively. The patient's infection indicators gradually declined, the vascular nevus progressively darkened and reduced in size, and symptoms showed gradual improvement. |
| 33 | Yu [9] et al. | 2023 | male | 67 | Right lung adenocarcinoma, stage IV | Pemetrexed + Carboplatin combined with Camrelizumab 200 mg | One cycle of camrelizumab administration | Nose tip, right upper eyelid, scalp area | no | The vascular nevus gradually darkens in color. |
| 34 | Yu [9] et al. | 2023 | male | 75 | Left lung squamous cell carcinoma, stage IIB | Paclitaxel (albumin-bound) + Carboplatin combined with camrelizumab 200 mg | Camrelizumab administration for over one month | Face and trunk | no | No specific treatment was administered; new vascular nevus was observed during follow-up two months later. |
| 35 | Jie[44] et al. | 2023 | female | 50 | Left lung adenocarcinoma, stage IVB | Pemetrexed 786 mg d1 combined with camrelizumab 200 mg d1 | After the second cycle of camrelizumab treatment | Left breast | yes | No specific treatment was administered; RCCEP resolved spontaneously. |
| 36 | Hui [45] et al. | 2024 | female | 49 | Acute myeloid leukemia type M2 | Camrelizumab 200mg | 13 days after administration of camrelizumab | Neck, chest, and back | no | No specific treatment was administered; RCCEP resolved spontaneously. |
| 37 | Zhou[7] et al. | 2024 | female | 44 | Cervical carcinoma | SHR-1210 200mg q3w | One week after initial administration of SHR-1210 treatment | Eyelid margin, forehead, and scalp | yes | No specific treatment was administered; RCCEP resolved spontaneously. |
| 38 | Zheng[46] et al. | 2024 | female | 58 | Cervical squamous cell carcinoma stage III | Camrelizumab 200mg | Approximately 3 weeks after the first injection of camrelizumab | Head and face, lower lip, trunk, and limbs | yes | Camrelizumab was discontinued, and the lip nodules were surgically excised with observation of remaining skin lesions; two months after discontinuation, the lesions began to regress spontaneously. |
| 39 | Tan[47] et al. | 2025 | male | 50 | Stage IV gastric cancer | Camrelizumab 200 mg d1 + albumin-bound paclitaxel 200 mg d1, d8, q21d, chemotherapy combined with immunotherapy for 7 cycles; subsequently switched to furquintinib 3 mg qd + camrelizumab 200 mg d1 for combined therapy for 9 cycles. | After 18 cycles of camrelizumab administration | Ascending colon, transverse colon, descending colon | no | Ceritinib was suspended, and methylprednisolone was administered for anti-inflammatory treatment, along with compound glutamine to repair intestinal mucosa and paracetamol-dihydrocodeine tablets for analgesia. The patient's abdominal pain alleviated without recurrence. |
| 40 | Huang[48] et al. | 2025 | male | 61 | Primary liver cancer | Apatinib combined with camrelizumab | After 9 cycles of camrelizumab administration | Concha nasalis inferior | yes | Local anesthesia was used for surgical resection; follow-up for 12 months showed no recurrence. |
| 41 | Huang[48] et al. | 2025 | male | 31 | Nasopharyngeal darcinoma | Albumin-bound paclitaxel combined with camrelizumab | After 9 cycles of camrelizumab administration | Pars nasalis pharyngis | yes | General anesthesia was used for surgical resection; follow-up for 12 months showed no recurrence. |
| 42 | Huang[48] et al. | 2025 | female | 28 | Nasopharyngeal darcinoma | Nintopuzumab + albumin-bound paclitaxel + cisplatin combined with camrelizumab | After 15 cycles of camrelizumab administration | Pars nasalis pharyngis | yes | General anesthesia was used for surgical resection; follow-up for 12 months showed no recurrence. |
| 43 | Huang[48] et al. | 2025 | female | 38 | Nasopharyngeal darcinoma | Camrelizumab, combination therapy with unknown drugs | After two cycles of camrelizumab administration | Nasal septum | yes | General anesthesia was used for surgical resection; follow-up for 6 months showed no recurrence. |
| 44 | Huang[48] et al. | 2025 | male | 21 | Nasopharyngeal darcinoma | Docetaxel + cisplatin combined with camrelizumab | After 14 cycles of camrelizumab administration | Nasal septum, nasal floor | yes | General anesthesia was used for surgical resection; follow-up for 8 months showed no recurrence. |

**Table 3 RCCEP Grading Criteria and Treatment Recommendations**

| Classify | Clinical manifestation | Treatment recommendation |
| --- | --- | --- |
| Level 1 | Single or multiple nodules with a maximum diameter ≤10 mm, with or without ulceration and bleeding | Continue medication administration. Apply gauze protection to areas prone to friction to prevent bleeding. For cases with ulceration and bleeding, local compression therapy may be employed for hemostasis. |
| Level 2 | Single or multiple nodules with a maximum diameter>10 mm, with or without ulceration and bleeding | Continue medication administration. Apply gauze protection to areas prone to friction to prevent bleeding. For ulcerated bleeding, local compression hemostasis may be employed, or local therapeutic interventions such as laser therapy or surgical excision may be considered. Prevent infection at the ulcerated site. |
| Level 3 | The condition is generalized and may be complicated by infection, potentially requiring hospitalization. | Discontinue medication until the condition is restored to grade ≤1 before resuming administration. Apply gauze protection to areas prone to friction to prevent bleeding. For cases with ulceration and bleeding, local compression may be employed for hemostasis, or local therapeutic interventions such as laser therapy or surgical excision may be considered. Concurrent infections require antimicrobial therapy. |
| Level 4 | Multiple and generalized manifestations, life-threatening | Discontinue medication permanently and seek immediate medical attention |
| Level 5 | die |  |
